# About a submucosal tracheal tumor

**DOI:** 10.1186/1477-7819-11-229

**Published:** 2013-09-14

**Authors:** Mounia Serraj, Marouane Lakranbi, Jamal Ghalimi, Yassine Ouadnouni, Siham Tizniti, Mohamed Smahi

**Affiliations:** 1 Faculty of Medicine and Pharmacy, Sidi Mohamed Ben Abdellah University, BP 1893. Km 2.200, Route Sidi Harazem, Fez 30000, Morocco; 2 Department of Respiratory Diseases, University Hospital Hassan II, Route Sidi Harazem, Fez, Morocco; 3 Department of Thoracic Surgery, University Hospital Hassan II, Route Sidi Harazem, Fez, Morocoo; 4 Department of Radiology, University Hospital Hassan II, Route Sidi Harazem, Fez, Morocco

**Keywords:** Trachea, Submucosal glands tumors, Mucoepidermoid carcinoma, Tracheal resection, End-to-end anastomosis

## Abstract

The authors report the case of 46-year-old man with recurrent hemoptysis. Bronchoscopy revealed a submucosal tumor protruding into the tracheal lumen. Transbronchial biopsy failed to obtain a conclusive diagnosis; only surgery allowed complete resection of the tumor and confirmed the diagnosis of tracheal mucoepidermoid carcinoma. We discuss the unusual submucosal presentation of this tumor, and the contribution of surgery for diagnosis and therapy.

## Background

Tracheal mucoepidermoid carcinoma (MEC) is a rare airway tumor and represents, along with the adenoid cystic carcinoma (ACC), one of the two most common types of primary salivary-type tracheal tumors. These tumors are indistinguishable histologically from their salivary gland counterparts, and it is believed that they originate from the submucosal glands of the tracheobronchial tree and probably are related to structural homology between exocrine glands. MEC can be divided into low grade and high grade on the basis of histological criteria. The most important factors in the prognosis include histological grading and the ability to achieve a complete surgical resection [1].

We report a case of a 46-year-old, previously healthy man, with a submucosal tracheal tumor, in whom transbronchial biopsies were inconclusive; only surgery allowed removal of the tumor and confirmed the diagnosis of tracheal MEC. To our knowledge, such a presentation of this tumor has not been previously reported.

## Case presentation

A 46-year-old male smoker presented with a 2-year history of hemoptysis. On admission, routine laboratory studies including pulmonary function tests were normal.

**Figure 1:**
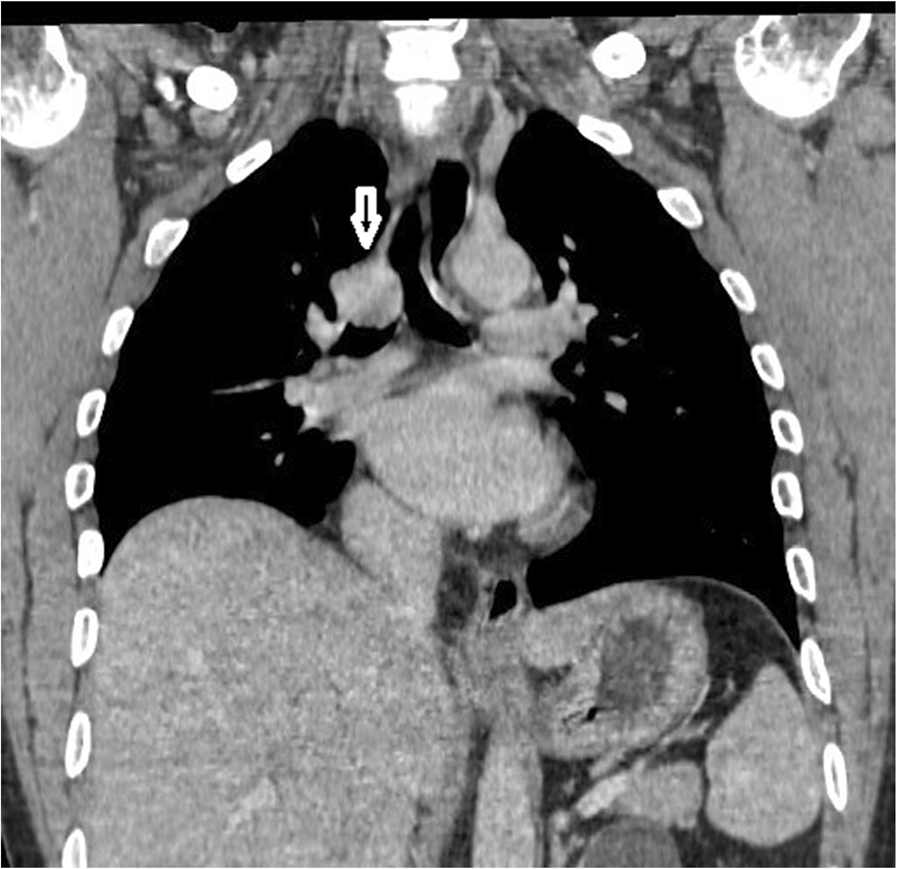
Computed tomography showed a circular mass in the lower trachea (arrow), with extra tracheal development.

**Figure 2:**
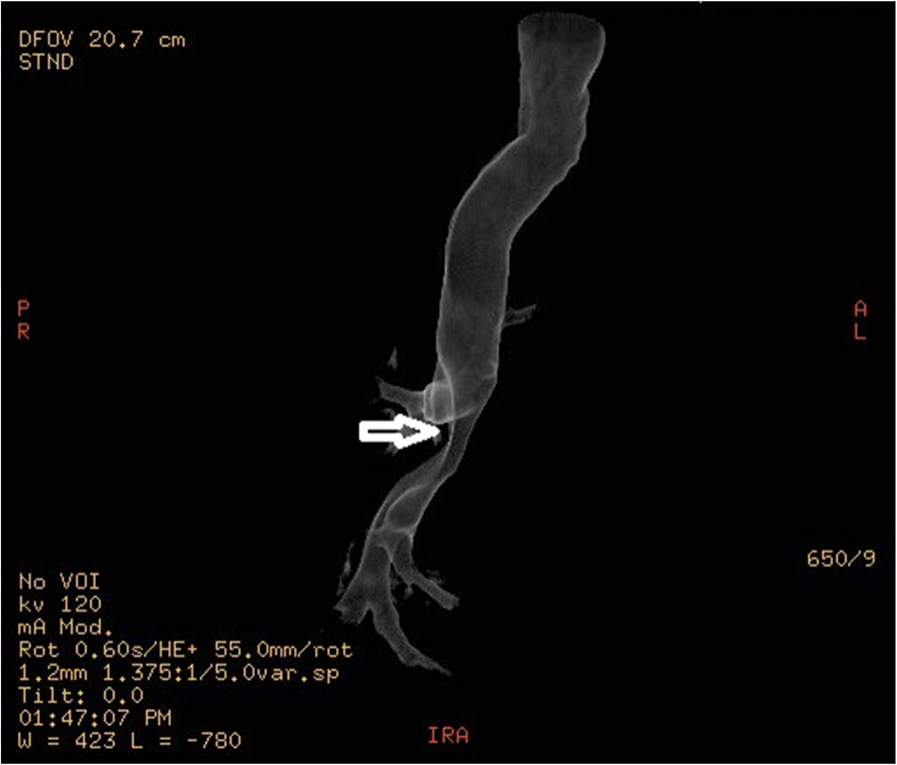
Spiral computed tomography showed that the tumor extended just across the carina (arrow).

The chest radiograph was normal. Computed tomography (CT) revealed a 4 × 4 × 3 cm circular mass in the lower third of the trachea, including the carina and repulsing the arch of azygous vein (Figure 1). There was no mediastinal lymphadenopathy or obvious findings of bronchial invasion. Spiral CT with multiplanar and three-dimensional reconstructions predicted the tumor extended to the origin of the right main bronchus (Figure 2). After further workup, there was no evidence of any metastatic lesion.

**Figure 3:**
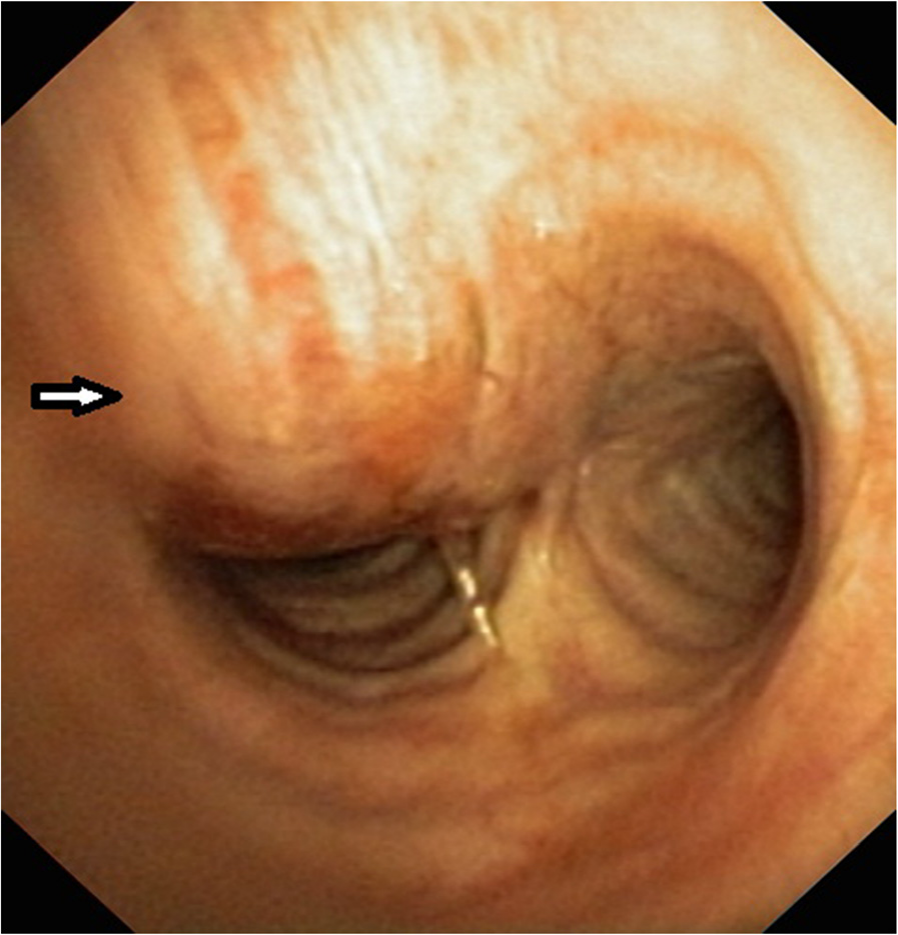
Fiberoptic bronchoscopy showed a submucosal tracheal tumor (arrow).

A flexible bronchoscopy showed a normal tracheal lumen, with bulging and erythematous mucosa, on the right posterolateral wall above the carina (Figure 3). Transbronchial biopsies were inconclusive.

**Figure 4:**
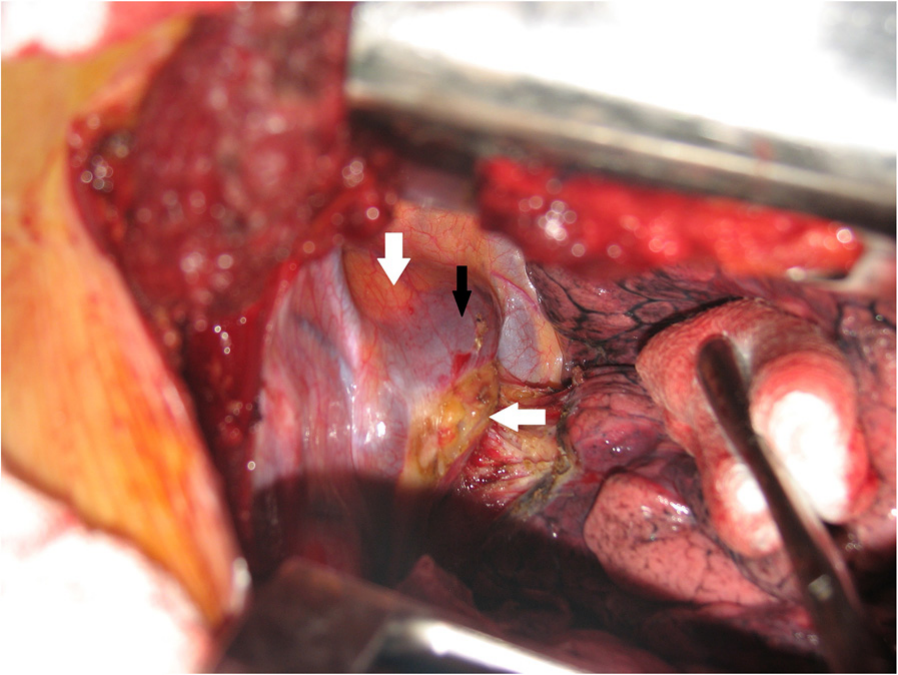
Intraoperative view showing the tumor (white arrows) displacing the arch of the azygous vein (black arrow).

**Figure 5:**
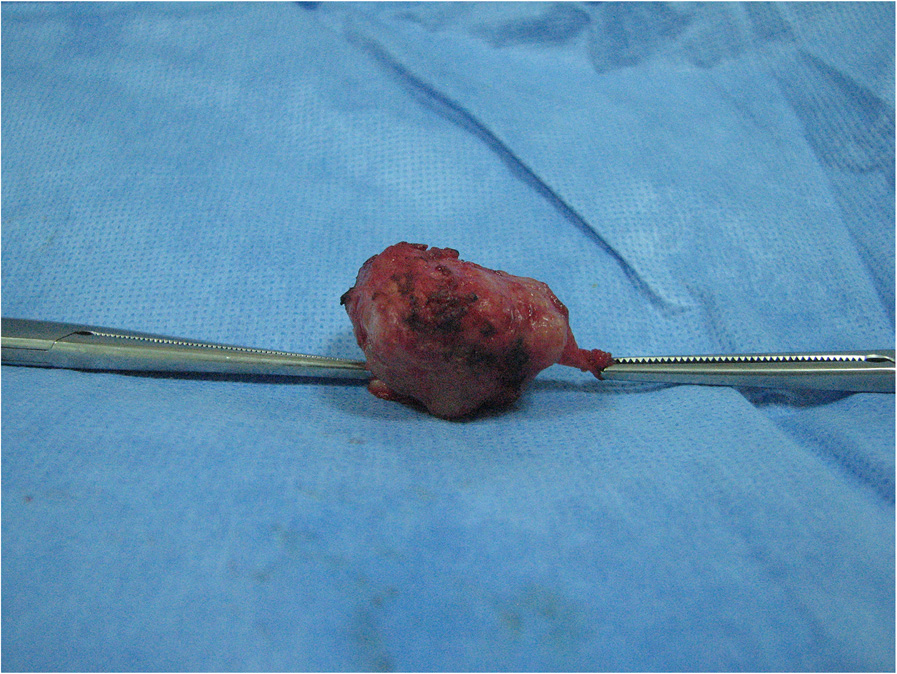
Photograph of the gross pathologic specimen obtained by angular resection and end-to-end anastomosis of the trachea, showing a well-circumscribed tumor.

**Figure 6:**
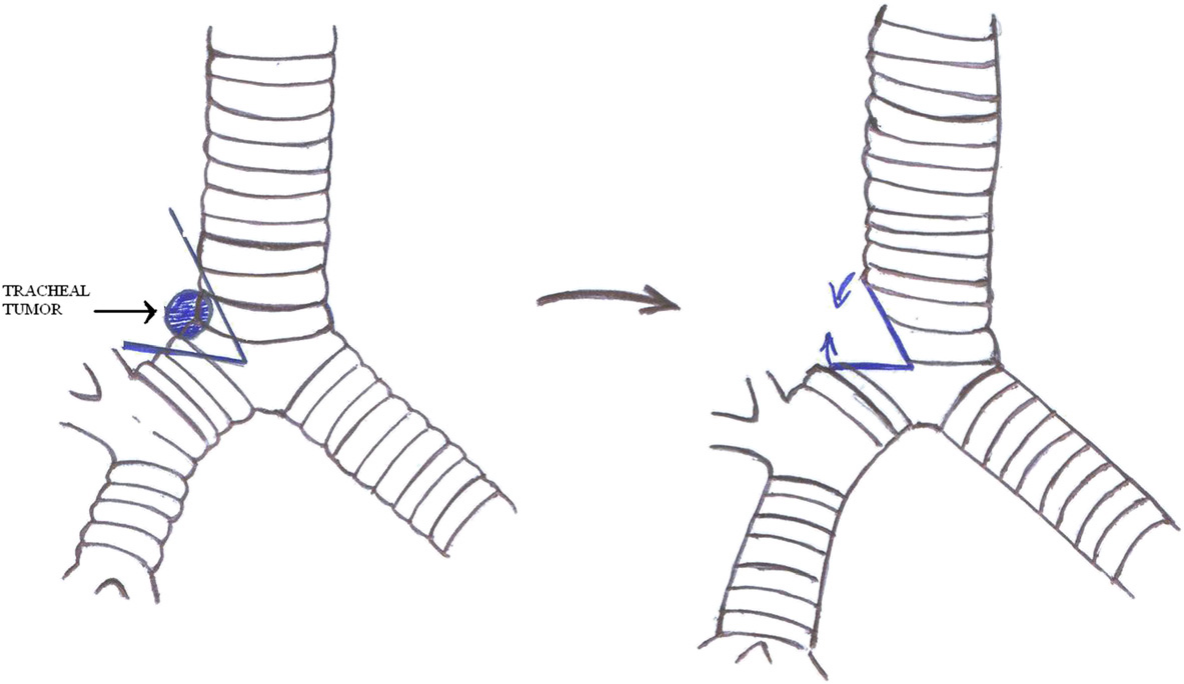
Diagram illustrating the tracheal resection and anastomosis performed.

Through a right posterolateral thoracotomy (Figure 4), the azygous vein was transected and a paratracheal and subcarinal lymph node dissection was performed. The tumor appeared to be at the expense of the tracheal wall. Complete excision of the mass (Figure 5) was performed by lateral resection of the trachea and reconstruction was made by end-to-end anastomosis with interrupted sutures on the cartilaginous part and posterior membrane of the trachea, using absorbable 4.0 polydioxanone sutures, without lung resection (Figure 6). Tracheal anastomosis was buttressed by a pleural flap. Intraoperative frozen examination revealed a mucoepidermoid tumor of the trachea with tumor-free surgical margins.

The postoperative course was uncomplicated; chest drain was removed on the fourth day following surgery. The final pathologic examination confirmed the diagnosis of primary low-grade MEC originating broadly from the trachea, without the involvement of the surgical margins. No evidence of metastatic MEC was identified in the lymph nodes that were excised. Follow-up 1 year after surgery is normal and the patient is today tumor free.

## Discussion

Tracheal MEC arises from the submucosal glands of the tracheobronchial tree and probably is related to structure homology between exocrine glands. It is a rare airway tumor, representing 0.2% of all respiratory tumors and 1 to 5% of ‘bronchial adenomas’ , a term that was used to describe a group of slow-growing neoplasms thought to arise from the bronchial glands, and included ACC, MEC, mixed tumors and carcinoid tumors [2]. However, it is clear that the group of salivary-type tracheal cancers, dominated by ACC and MEC, comprise a distinct category of tracheal neoplasms.

Histologically, MEC of the tracheobronchial tree consists of variable proportions of mucus secreting cells, squamous cells, and so-called intermediate cells that show no particular differentiating characteristics [2]. The tumor is histopathologically classified as a low- or high-grade malignancy. Mitoses, nuclear pleomorphism, and necrosis are usually absent or minimal (less than five mitoses per 50 high-power fields) in low-grade MEC [2]. In high-grade tumors mitoses are increased, averaging four per 10 high-power fields, and nuclear pleomorphism, hyperchromasia, and cellular necrosis are present.

Generally, these tumors produce symptoms of upper respiratory tract obstruction, such as cough, dyspnea, hemoptysis, wheezing, atelectasis or postobstructive pneumonia, and it frequently is mistaken for asthma or chronic pulmonary obstructive disease, with no or partial response to treatment. When the symptoms are not resolved, a more scrupulous workup is usually performed and intratracheal tumors diagnosed.

Imaging studies usually precede bronchoscopy, unless the patient arrives in respiratory distress. Improvements in technology now allow CT scanning to provide various images for airway visualization. As far as diseases involving the central airways are concerned, the transverse extent of disease and its relationship to adjacent structures are better shown on the usual transverse CT sections, but the longitudinal extent of the tumor is better demonstrated on the multiplanar reconstruction and three-dimensional images [3]. In the present case, the CT has been of great help in visualizing the extraluminal portion of the tumor, allowing us to better prepare the surgical approach. Patients with malignant tumors undergo a search for metastatic disease that typically includes examinations of the lung, brain, bone, adrenal glands, and liver.

Every patient suspected of having a tracheal tumor should undergo bronchoscopy with biopsy, which remains the main diagnostic modality in nonemergency cases. MEC often presents as a sessile tracheal tumor.

In the present case, the MEC had an unusual submucosal presentation, explaining the negative bronchial biopsies and hence the need for surgery for therapeutic and diagnostic purposes. This surgery requires an accurate preoperative assessment, including a bronchoscopy and chest CT with multiplanar tracheal reconstructions. The usual recommendations of tracheal surgery must be respected, from the induction of anesthesia until the resection-anastomosis, without omitting the intraoperative ventilation technique. A biopsy with frozen section examination must be performed because the operative strategy would be affected by the results.

The clinical course of these tumors correlates with the histological grade of the tumor. Patients with high-grade lesions have been reported to have a poor prognosis; the 5-year survival rate is 25 to 31% [4]. Low-grade tumors present localized growth, rarely affect the lymph nodes and are easily resected, with a normal life expectancy, and 5-year survival after resection can be as high as 80% [5]. Lymph node involvement has been reported to be an indicator of a worse prognosis; only 2% of low-grade tumors and 15% of high-grade tumors metastasized to the regional lymph nodes. ACC has a higher likelihood than MEC to metastasize. Overall, patients with MEC survive better than patients with ACC.

Complete resection of the tumor on-bloc with the tracheal rings combined with reconstruction of the trachea is the mainstay of treatment. The role of radiotherapy and chemotherapy, before or after surgery, has yet to be well established [6].

## Conclusion

In summary, a tracheal MEC may be submucosal, and the transbronchial biopsy may remain negative. In this case, surgical excision for diagnostic and therapeutic purposes is possible, after performing CT with multiplanar reconstructions.

## Consent

Written informed consent was obtained from the patient for the study and publication of this case report and accompanying images. A copy of written consent is available for review from the Editor-in-Chief.

## Abbreviations

ACC: Adenoid cystic carcinoma; CT: Computed tomography; MEC: Mucoepidermoid carcinoma.

## Competing interests

The authors declare that they have no competing interests.

## Authors’ contributions

SM collected information and prepared the original draft. LM and GJ researched the relevant literature and revised the draft. OY helped with the literature research and preparing the manuscript. SM helped prepare the manuscript. All authors read and approved the final manuscript.
